# Daily Work-Family Conflict and Burnout to Explain the Leaving Intentions and Vitality Levels of Healthcare Workers: Interactive Effects Using an Experience-Sampling Method

**DOI:** 10.3390/ijerph18041932

**Published:** 2021-02-17

**Authors:** Luis Manuel Blanco-Donoso, Jennifer Moreno-Jiménez, Mercedes Hernández-Hurtado, José Luis Cifri-Gavela, Stephen Jacobs, Eva Garrosa

**Affiliations:** 1 Faculty of Psychology, Autonomous University of Madrid, 28049 Madrid, Spain; jennifer.moreno@uam.es (J.M.-J.); mercedes.hernandezh@estudiante.uam.es (M.H.-H.); jose.cifri@estudiante.uam.es (J.L.C.-G.); eva.garrosa@uam.es (E.G.); 2 The School of Nursing, University of Auckland, Auckland 1142, New Zealand; s.jacobs@auckland.ac.nz

**Keywords:** work-family conflict, burnout, leaving intentions, vitality, diary study, experience-sampling method, healthcare workers

## Abstract

There is an intensification of work in global health systems, a phenomenon that could increase work-family conflict, exhaustion, and intentions to leave among healthcare workers. The main objective of this study is to analyze if daily work-family conflict and burnout could explain the daily leaving intentions and vitality of healthcare workers. This is a diary study, which employs an experience-sampling methodology (ESM). A total of 56 physicians, nurses, and nursing aides from intensive care and nephrology units filled out various quantitative scales during 5 working days (56 × 5 = 280 observations). Multilevel hierarchical analysis showed that daily work-family conflict and burnout were significantly associated with higher daily intentions of leaving the profession, and with lower levels of daily vitality. In addition, those workers who experienced more work-family conflict and depersonalization on a daily basis were those who showed more intentions to leave and less daily vitality, showing an interactive effect. The results highlight the importance of examining the psychosocial risks experienced by healthcare workers by employing experience-sampling methodologies, which could help us to deepen our understanding of the proximal antecedents of their intentions to leave and their psychological well-being.

## 1. Introduction

In recent years, work intensification has increased considerably, especially among healthcare professions [1,2]. This situation could have been further aggravated during the crisis caused by COVID-19 [3], particularly among those professionals working in an Intensive Care Unit (ICU), and in other highly specialized units, such as dialysis units [4,5,6].

Working in these types of medical units involves dealing with complex and high physical, cognitive and emotional demands [7,8,9]. Therefore, these workers are exposed to multiple psychosocial risks such as job stress, burnout, emotional labour, workplace aggressions, or work-family conflict, among others [10,11,12,13]. In turn, these risks have been associated with poorer physical and mental health [14,15], reduced psychological well-being [16], and lower quality of care [8,17,18]. These risks also interfere with workers’ private lives, as well as their family environment, hindering them from satisfactorily balancing both areas of their lives [2]. Ultimately, the presence of these psychosocial risks can increase workers’ leaving intentions, which may mean either changing the medical unit for another care service, or permanently leaving their profession [19,20].

High rates of turnover of health professionals are damaging for the workers themselves, but also for health organizations and society in general [20]. Nowadays, health systems suffer from a lack of personnel to respond adequately to the health demands of the population, which hinders countries from effectively fighting against disease or promoting health among their citizens [21]. Turnover is likely to increase workload and levels of stress among current workers, while also diminishing the quality of care provided to the patients they are taking care of. For this reason, many studies have focused on factors related to health professional turnover to develop interventions aimed at preventing them from leaving [20,22,23,24]. Measurement of their intentions to leave shows that the final decision of a health professional to leave the profession can be predicted [25], therefore intervening on the risk factors associated with intention to leave may prevent this negative outcome from happening [26].

From this perspective, various cross-sectional and longitudinal studies have revealed how different variables such as work stress, overload, conflicts within the team, opportunities for professional growth, job satisfaction, burnout, or work-family conflict are associated with a higher rate of leaving intentions [20,22,23,24,27,28]. However, as far as we know, few studies have explored the antecedents of leaving intentions using an experience-sampling methodology and a diary design. The experience sampling methodology (ESM) includes all data collection methods that capture participants’ behavior and experiences repeatedly over a short period of time, e.g., diaries for five or ten days. The aim of the ESM studies is to capture short-term episodic changes between mechanisms that affect each other rapidly, rather than long-term changes that occur over months or years. The ESM design (1) provides insights into how multiple dynamic changes influence outcomes, (2) enhances ecological validity, (3) allows researchers to examine both within and between-person processes, and (4) reduces retrospective recall biases [29].

In this regard, it would be worth studying how intentions to leave the profession might fluctuate over days or weeks, depending on temporary variations in the risk factors associated with those intentions [30,31]. For example, a recent diary study conducted by Shi et al. [30] with 65 hotel workers shows that intentions to leave the job fluctuate, and these fluctuations can be explained by the daily variations in the emotional dissonance workers experience, as well as by their daily levels of autonomy at work. Therefore, leaving intention can vary over short periods. Specifically, the results obtained by Shi et al. [30] showed that the daily variability within the person regarding their intention to leave was 41.84%, indicating that a greater percentage of the variability of this variable was due to daily differences within the person. Similarly, healthcare professionals may also want to quit their jobs more frequently on those days in which they have experienced certain stressors, or when certain resources have not been present [32,33]. However, this hypothesis has not been tested yet using a daily diary design and experience-sampling methodology. In line with this, some experts have recently highlighted a need to develop context-specific investigations of turnover intentions that can address its fluctuations [34].

The relationship between work-family conflict and leaving intention is well documented in the literature, as well as the relationship between work-family conflict and other psychological and occupational well-being indicators [35,36,37,38]. By work-family conflict we understand “a form of inter-role conflict in which the role pressures from the work and family domains are mutually incompatible in some respect” ([39] p. 77). Specifically, work-family conflict occurs when work-related activities make it difficult for a person to fulfill their family or personal responsibilities [40]. According to Greenhaus et al. [39], there are three major forms of work-family conflict: time-based conflict (i.e., time demands associated with one role make it difficult to meet the requirements of other roles); strain-based conflict (i.e., the feelings of irritability or tension produced by the performance of one role are transferred to other areas); and behavior-based conflict (i.e., the behaviors required for one activity are incompatible with the behaviors required for another activity). Later, Greenhaus et al. [41] extended their typology by adding an additional energy-based conflict (i.e., the energy used in the performance of one role diminishes the performance of another role).

The stress generated by work-family conflicts may lead workers to consider leaving their profession, their job or the organization they work for in order to resolve the conflict and reduce the stress they are currently experiencing [42]. However, this link between work-family conflict and leaving intention has not been tested yet over short periods of time or using a diary method, with most of the studies in this area being cross-sectional studies [29]. Studying the association between these two variables on a daily level implies accepting work-family conflict as a dynamic experience that can vary depending on certain contextual or personal variables, with consequences that also vary [29].

Burnout is a syndrome characterized by three main dimensions: emotional exhaustion, depersonalization and low personal accomplishment [43]. While the emotional exhaustion dimension refers to feelings of being overwhelmed and experiencing one’s emotional and physical resources being drained, the depersonalization dimension (also known as cynicism) is expressed as a poor, insensitive and overly impersonal response to different aspects of the job, especially those related to interpersonal relationships (e.g., patients, colleagues or supervisors) [44]. High levels of emotional exhaustion and depersonalization have been found among ICU health professionals [45,46], and both dimensions have been related to turnover intention [19,22,47,48]. However, the associations between these variables have been found through studies using cross-sectional and longitudinal methodologies, whereas exhaustion and depersonalization and their consequences can fluctuate on a daily basis [49,50].

Experiences of emotional exhaustion and depersonalization on a daily basis [13,51] may be key factors that increase health professionals’ intention to leave on days when work-family conflicts are also present. Experiencing work-family conflict results in higher levels of burnout (the mediating variable) leading to increased intention to leave [45]. However, the hypothesis that burnout is a moderating variable in the relationship between work-family conflicts and leaving intentions needs to be further explored.

Increased work-family conflict on a daily basis can affect health professionals’ leaving intentions, especially on those days when they have experienced greater emotional exhaustion or have had depersonalizing attitudes towards patients. These relationships can be explained using two different models: the conservation of resources (COR) model proposed by Hobfoll [52] to explain the interaction between work-family conflict and emotional exhaustion in explaining intention to leave; and the career change model proposed by Rhodes and Doering [53] to explain the interaction between work-family conflict and depersonalization.

According to the COR-model, individuals strive to acquire, accumulate and maintain those things and resources they value (e.g., material or psychological resources, energy…). From this perspective, stress occurs when these resources are threatened or lost. Thus, work-family conflict is understood in this model as a consequence of a loss of resources while trying to fulfill all their responsibilities in both work and family contexts [54]. Also, according to Hobfoll [55], emotional exhaustion can be understood as the result of a prolonged investment of resources in work (e.g., energy), without obtaining the expected and valued resources in return [55,56]. However, this model also highlights the fact that, when facing stressful situations, people can mobilize other resources still available to deal with current challenges and thereby recover from resource and well-being losses. From our point of view, if work-family conflict is present on a daily basis, and workers are also emotionally exhausted, it becomes increasingly difficult to recover well-being and reduce stress. Also, workers are more likely to attribute resource loss to their work, finding themselves exhausted in relation to their profession and developing a negative attitude towards their interpersonal relationships at work [57]. From this perspective, intention to leave the profession seem to be as a strategy to restore personal balance and lost resources [42,58,59].

Another model which helps make sense of workers’ intention to leave their profession is the career change model. This model states that the main motivation that drives career change is job or career dissatisfaction. According to Rhodes and Doering’s (1983) [53], work-family conflict may be one of the possible causes of this dissatisfaction. If career dissatisfaction and leaving intentions are greater on days when workers experience work-family conflict, resulting in emotional exhaustion and depersonalization, then these two burnout dimensions could increase professional dissatisfaction and workers’ leaving intentions.

To conclude, this study has also included subjective vitality as an outcome variable in order to test how the interaction between daily work-family conflict and burnout may have an impact on daily psychological well-being indicators. Ryan et al. ([60], p. 530) define subjective vitality as “one’s conscious experience of possessing energy and aliveness”. According to these authors, vitality concerns a specific psychological experience of possessing enthusiasm and spirit and people vary in their experience of vitality as a function not only of physical influences (e.g., states of fatigue), but also psychological factors (e.g., being in love or having a mission). To our knowledge, there is a lack of studies concerning vitality and the relationships with burnout and work-family conflict. Despite this, research focused on healthcare professionals revealed that emotional demands required for these professions were associated with subjective vitality when personal or job resources are available [61,62]. In one hand, it is well-supported that these emotional demands could affect the balance work-family [63], as the excess of demands at work might lead an inter-role conflict [64], and in turn, impact on subjective vitality levels. On the other hand, burnout is considered as a negative outcome derived from the increase in job demands when personal/job resources are not enough to accomplish them [43], generally negatively related to vitality in healthcare contexts [65]. In this regard, identifying the daily and short-term antecedents of vitality is especially relevant [61,66], since healthcare professionals are currently experiencing high levels of exhaustion and fatigue [4,67,68]. In addition, high levels of subjective vitality and energy have been associated with greater proactivity at work, goal attainment [69], organizational citizenship behavior [70], and with a better job performance [71].

**Figure 1:**
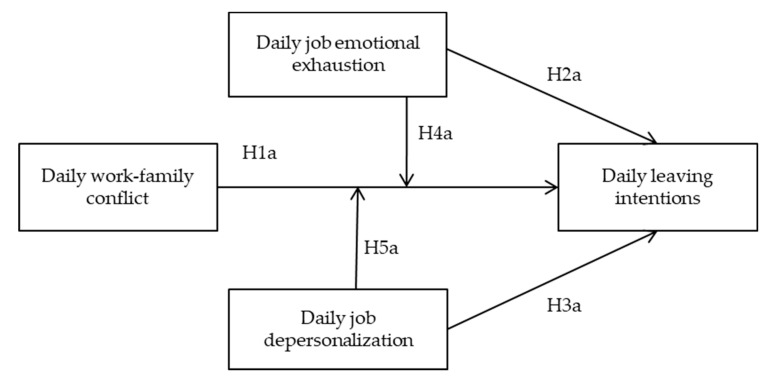
Research model and hypotheses to explain daily leaving intentions.

**Figure 2:**
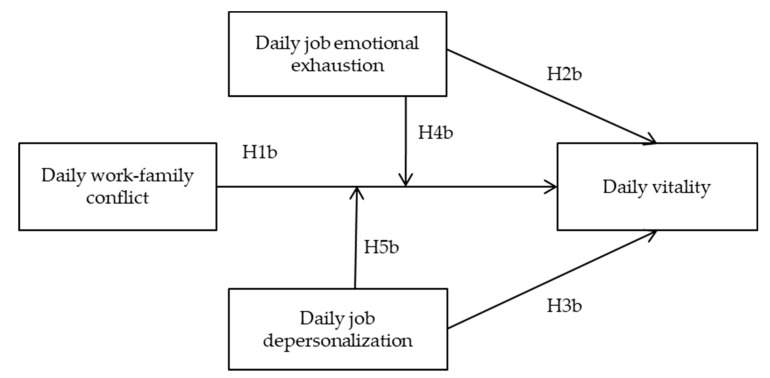
Research model and hypotheses to explain daily vitality levels.

In short, this study had three objectives. Firstly, to explore predictors of intention to leave among a group of healthcare workers on a daily basis, and specifically to explore the role daily work-family conflict and burnout has on the intention to leave the profession of these professionals. Secondly, to explore if daily emotional exhaustion and depersonalization boost the relationship between work-family conflict and daily leaving intentions. Finally, to test if the relationships and predictions mentioned above about workers’ leaving intention can be extended to psychological well-being indicators, specifically, to daily levels of subjective vitality. Thus, the following hypotheses were formulated and the hypothesized model can be seen in Figure 1 and Figure 2:

**Hypothesis** **1 (H1).** *Daily work-family conflict will be significantly and (1a) positively related to daily leaving intention and (1b) negatively related to daily vitality, before go to bed.*

**Hypothesis** **2 (H2).** *Daily job emotional exhaustion will be significantly and (2a) positively related to leaving intention and (2b) negatively related to daily vitality, before go to bed.*

**Hypothesis** **3 (H3).** *Daily job depersonalization will be significantly and (3a) positively related to leaving intention and (3b) negatively related to daily vitality, before go to bed.*

**Hypothesis** **4 (H4).** *Daily work-family conflict will be more positively related to leaving intentions (4a) on days where healthcare workers feel more job emotional exhaustion as opposed to days where they feel less job emotional exhaustion; and (4b) on days where healthcare workers feel more depersonalization as opposed on days where they feel less job depersonalization.*

**Hypothesis** **5 (H5).** *Daily work-family conflict will be more negatively associated with daily vitality (5a) on days where healthcare workers feel more job emotional exhaustion as opposed on days where they feel less job emotional exhaustion; and (5b) on days where healthcare workers feel more depersonalization as opposed on days where they feel less job depersonalization.*

## 2. Materials and Methods

### 2.1. Participants and Procedure

In this study, one hundred healthcare professionals working in the ICU and nephrology units of four Spanish public hospitals were invited to participate. Three of the four hospitals belong to the same province as the researchers. These hospitals have been collaborating regularly with the research team in different research and teaching objectives. Once contacted with the supervisors of these units, the researchers proposed to participate in the study to the workers within their unit. Each worker received two surveys: a general one to evaluate the general measure of the variables of this study; and a daily one, to evaluate the same variables for 5 days adapted to the daily evaluation. Firstly, they had to fill in the general questionnaire and subsequently, they had to complete daily questionnaires (paper-and-pencil surveys), one time a day (before going to bed), for five consecutive working days. During the study, researchers regularly contacted the participants and supervisors to sustain their participation. All participants also received a letter explaining the objectives of the study and a form to be signed as a written informed consent. Workers then returned the questionnaires in a sealed envelope to the researchers, by mail or directly at a personal meeting. The study protocol was approved by the Ethical Committee of the (masked for review) (CEI-83-1545). To guarantee confidentiality, responses were matched using anonymous codes.

This research was conducted prior to the COVID-19 pandemic. Of the 100 surveys distributed, 56 were returned (response rate = 56%; 80.4% women, 19.6% men) from ICU (*n* = 46), and nephrology units (*n* = 10). The sample consisted of physicians (*n* = 24), nurses (*n* = 22), and nurse aides (*n* = 9) (one missing value). Their educational qualifications were respectively a medical degree, a nursing degree and an aide-nursing technical qualification. This is a considerable sample for a diary study (*N* = 56 × 5 days = 280 observations), and this sample size met the minimum number of 30 proposed by Scherbaum & Ferreter (2009) [72] for diary studies and multilevel analysis. The mean age was 40.05 years (SD = 11.49), and the majority of the participants lived with a partner (85.7%). 48.3% of all participants had children. The average working hours per week was 41.04 h (SD = 10.65), the average years of work experience was 15.34 (SD = 9.63), and the average years of tenure in their work centers was 9.33 (SD = 8.39). Eight of them were supervisors.

### 2.2. Variables and Instruments

Daily measures of all variables used modifications of items from the corresponding general-scale, which was reworded for daily administration. Moreover, for daily measures we used the same answer categories as for the general measure. This method of developing state-level analogs of general measures has been used successfully in the past [73]. All the questionnaires included in this study have been validated in Spanish and these adapted versions were used in this study.

Work-family conflict was assessed using the Negative Work-family Interaction Subscale of the Nijmegen Work-Home Interaction Survey (SWING) [74,75]. For the daily measure, the items were adjusted so that they referred to the preceding workday (e.g., “Today, I’ve been irritated at home because my work is so exhausting” or “Today, I have had to work so hard that I haven’t had time for my hobbies”). Each item was rated on a 4-point Likert-type scale, ranging from 0 (totally disagree) to 3 (totally agree). SWING has been found to have adequate reliability and validity [70,71]. In this study, Cronbach’s alpha ranged from 0.73 to 0.84 (M = 0.79) for the daily measure.

Emotional exhaustion and Depersonalization were assessed using the Nursing Burnout Scale (NBS) [76]. For the daily measure, the items were adjusted so that they referred to the preceding workday (e.g., “Today, I felt exhausted at the end of workday” for exhaustion or “Today, I tried to depersonalize as much as possible the relationship with the patients’ relatives, and when I could I avoided contact with them” for depersonalization). Each item was rated on a 4-point Likert-type scale, ranging from 1 (totally disagree) to 4 (totally agree). The NBS has been found to have adequate reliability and validity [77]. In this study, Cronbach’s alpha for emotional exhaustion ranged from 0.88 to 0.90 (M = 0.89) for the daily measure, and from 0.79 to 0.89 (M = 0.85) for depersonalization. The items of these subscales are worded in such a way that it is possible to assess both physicians and nurses.

Leaving intentions were assessed using the Professional Consequence Subscale of the Nursing Burnout Scale (NBS) [76]. For the daily measure, the items were adjusted so that they referred to the preceding workday (e.g., “Today, I want to leave the profession” or “Today, I would like to change my profession”). Each item was rated on a 4-point Likert-type scale, ranging from 1 (totally disagree) to 4 (totally agree). The NBS has been found to have adequate reliability and validity [77]. In this study, Cronbach’s alpha for the general measure was α = 0.91, and it ranged from 0.86 to 0.92 (M = 0.89) for the daily measure. As in the above case, the items of this subscale also allow the assessment of physicians and nurses.

Subjective vitality was measured with the Ryan and Frederick’s Vitality Scale [60,78]. This scale assessed the degree to which participants felt physically and mentally vigorous and alert in every domains. Daily vitality was measured with the scale modified so that the items referred to the present moment. An example item is: “At this moment, I feel alive and vital”. The Vitality Scale has been found to have adequate reliability and validity [60,78]. In this study, Cronbach’s alpha for the general measure was α = 0.87, and it ranged from 0.79 to 0.87 (M = 0.83) for the daily measure.

### 2.3. Statistical Analysis

Hierarchical linear modeling was used to test our hypotheses because the collected data included variables from two levels, with days (Level 1; *N* = 280 study occasions) nested within individuals (Level 2; *N* = 56 participants). Data were analyzed using MLwiN 2.28 software [79]. Following Ohly et al. [80], we centered predictor variables at the person level around the grand mean (Level 2: sex, age, time, shift, and general measure of leaving intentions and subjective vitality); and predictor and outcome variables at the day level around the respective person mean (Level 1: daily work-family conflict, daily emotional exhaustion, daily depersonalization, daily leaving intentions and daily subjective vitality). Due to previous associations found in the literature between sociodemographic variables (i.e., sex and age) and the variables of this study [20,38], sociodemographic variables (together with the shift) were introduced in the first step to control for their possible confounding effects. To control for the possible effect of profession, we generated a dichotomous variable (1 = physician; 2 = nursing workers) and included it in the multilevel analyses. Additionally, we included the variable “time” in the analyses as a control variable in order to control for possible accumulation effects in the dependent variable over the course of the five working days. In addition, the baseline levels of the outcome measures are included in the models as controls. Including baseline levels enables us to analyze the daily fluctuations around the baselines of workers, which is relevant due to the fact that employees’ general levels could affect their momentary states [81]. Thus, interpretations of our results based on stable differences between persons can be ruled out because we used person-level variables as control variables before entering day-level variables in subsequent models of analysis [73]. In this study, significant interactions indicated that the effect of daily work-family conflict on daily leaving intentions and subjective vitality depends on the Level 1 variables (i.e., daily emotional exhaustion and depersonalization). Simple slope tests were conducted following advice from Preacher et al. [82] for significant interactions.

## 3. Results

In order to examine the total variance at the within-person level, we estimated the intra-class correlation coefficient (see Table 1). Table 1 also shows the means, standard deviations, Cronbach’s alphas and correlations among all the study variables.

### 3.1. Hypothesis Testing

Table 2 and Table 3 show the multilevel models in predicting daily leaving intentions (Table 2) and daily subjective vitality (Table 3). Concerning the sociodemographic variables (control variables), we found a significant effect of age (B = 0.017, SE = 0.007, t = −2.428, *p* < 0.05), and time (B = 0.030, SE = 0.013, t = 2.307, *p* < 0.05) for daily leaving intentions, whereas the variable “profession” showed a significant effect on daily subjective vitality (B = −0.532, SE = 0.195, t = −2.728, *p* < 0.001).

Following the research model proposed in Figure 1 and Figure 2, we found statistical support for H1a and H1b (B = 0.169, SE = 0.042, t = 4.024, *p* < 0.01, and B = −0.446, SE = 0.087, t = −5.126, *p* < 0.001, respectively), that is, daily work-family conflict appeared to be a positive predictor for daily leaving intentions and a negative predictor for daily vitality. Regarding H2a and H2b, we found statistical support for both, resulting in daily job emotional exhaustion being a positive predictor for daily leaving intentions (B = 0.153, SE = 0.056, t = 2.732, *p* < 0.01), and a negative predictor for daily vitality (B = −0.431, SE = 0.116, t = −3.715, *p* < 0.001). Regarding daily depersonalization, we only corroborated H3a, hence daily depersonalization appeared to be a positive predictor for daily leaving intentions (B = 0.124, SE = 0.061, t = 2.033, *p* < 0.01), but not for daily vitality (B = 0.058, SE = 0.125, t = 0.464, *p* > 0.05).

### 3.2. Interaction Effects

**Figure 3:**
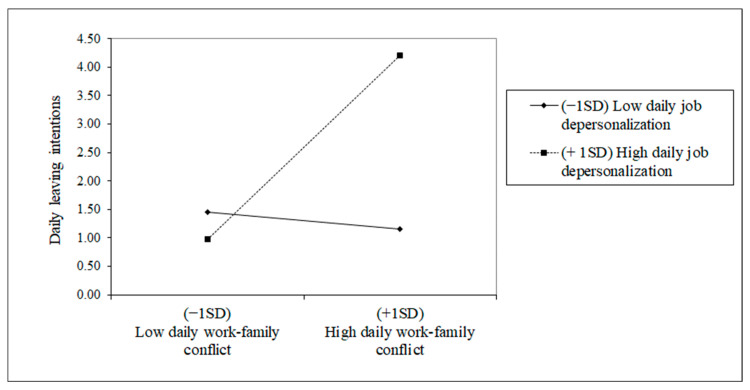
Interaction effects of Daily work-family conflict and Daily job depersonalization in explaining Daily leaving intentions.

Examining the interaction effects previously proposed, we observe model 4 in Table 2 and Table 3. Concretely, neither interaction effect regarding daily job emotional exhaustion seems to be significant (B = 0.028, SE = 0.104, t = 0.269, *p* > 0.05, and B = 0.075, SE = 0.210, t = 0.357, *p* > 0.05). However, we found a significant interaction effect concerning daily job depersonalization. Firstly, we corroborated H4a as we found a significant interaction effect of daily depersonalization between daily work-family conflict and daily leaving intentions (B = 0.251, SE = 0.103, t = 2.437, *p* < 0.01; see Table 2). As we can see in Figure 3, there is a boosting effect, finding through the simple slope test that daily work-family conflict is significantly and positively related to daily leaving intentions, especially when daily job depersonalization is high rather than low (y = 0.859, SE = 0.286, z = 2.998, *p* < 0.01).

**Figure 4:**
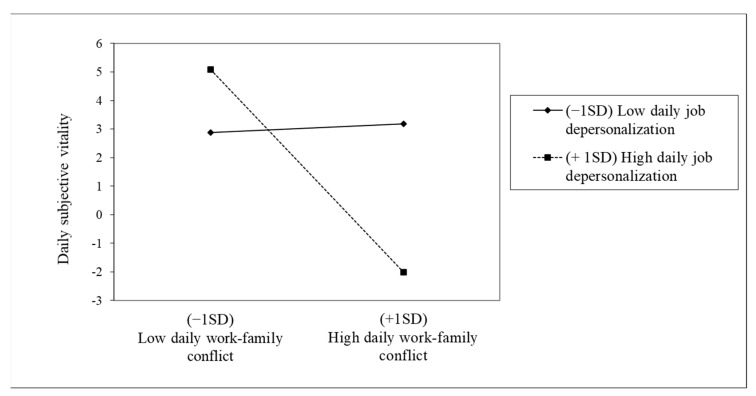
Interaction effects of Daily work-family conflict and Daily job depersonalization in explaining Daily subjective vitality.

On the other hand, we also found a significant interaction effect with daily depersonalization and work-family conflict predicting daily vitality (B = −0.526, SE = 0.211, t = −2.492, *p* < 0.01). Moreover, we encountered a boosting effect of this daily depersonalization between daily work-family conflict and daily vitality (see Figure 4), seeming to decrease daily vitality when depersonalization is higher. A simple slope test revealed that daily work-family conflict was significantly and negatively related to daily vitality especially when daily depersonalization is high rather than low (y = −1.892, SE = 0.603, z = −3.137, *p* < 0.01).

## 4. Discussion

The objective of this study was to analyze the daily impact of work-family conflict on intentions to leave the profession and the vitality levels of healthcare professionals on a daily level. Our study contributes to the literature as it (a) adds evidence on intra-individual variations of leaving intentions and vitality levels of healthcare workers; and (b) examines the short term psychosocial mechanisms (i.e., daily work-family conflict and burnout) that explain those daily leaving intentions and energy levels [29,30,34]. The results of this study show that work-family conflict is a stressor that determines on a daily basis any feelings of loss of relevant resources in health professionals, as proposed in the starting models, the COR model [52,55], and the career change model [53].

Work-family conflict is identified as a daily predictor of intention to leave the profession. Moreover, on days when healthcare professionals perceive high levels of work-family conflict and depersonalization, they have a high desire to leave their profession. Without a doubt, this highlights work-family conflict as a high-risk factor for the good functioning of the health system that health services need to be aware of. This study shows that conflict between work and personal life not only means an increase in the stress levels of professionals, but also a decrease in work motivation that can lead to the abandonment of the profession, as well as daily individual consequences for the individual through decreased levels of vitality. Work-family conflict is strongly related to burnout, loss of energy and vitality levels [83]. When health professionals are unable to balance their personal life and work, they have low vitality scores, as well as feelings of frustration at not being able to adequately manage both relevant spheres in their lives, which has implications for the performance of their work on a daily basis. Work-family conflict may also be causing difficulties in resting or relaxing, as well as difficulties in recovering from stress [61]. If work-family conflict is a friction that is produced between the pressures exerted by the work and by the family that are incompatible [39], leaving may be the only option available to the health professional to solve the conflict if health services management does not intervene to support the professional to find work related approaches to ease the stress. According to the COR-model, work-family conflict can arise because the worker has insufficient resources available to them to enable them to attend to all their responsibilities. Their unsuccessful efforts may lead to a drain on other resources, such as vitality, resulting in intention to leave the profession as a way to better manage the resources available to them (i.e., the balance between both spheres of life and more energy) [42,54,57,58,59].

The significant interaction found between work-family conflict and depersonalization and the impact on increased leaving intentions and decreased daily energy levels shows that these problems are especially acute on days when depersonalization is high. Feelings of depersonalization imply an important distancing from the professional role. When the stress derived from work-family conflict is added, it affects daily energy levels, diminishing vitality and increasing intention to abandon the profession. The career change model could explain these results. Those workers who find it difficult to balance their professional and personal lives on a daily basis, and who are also distant from patients and have negative attitudes towards them, could show more desire to leave because their profession does not seem to be giving them satisfaction. Working with patients in highly specialized units has a vocational component, and contributes to professional identity. When the work does not meet the worker’s expectations and generates dissatisfaction, the worker may decide to leave the profession, especially if the source of dissatisfaction is working with patients, a fundamental component of healthcare work. In addition, this dissatisfaction generated by the lack of balance between personal and professional life can also decrease levels of subjective vitality in workers [84].

This study, as mentioned, presents preliminary results in terms of the relationship between work-family conflict and leaving intention, identified for the first time over short periods of time using a diary method, health professional turnover and intention to leave has a high costs for both the workers themselves, but also for organizations and society in general [20]. Organizations that understand that a professional’s intention to leave varies daily and is influenced by factors such as work-family conflict can take timely preventive and organizational management preventative measures.

This study also corroborates that professionals in the ICU and nephrology units are subject to high stress situations as well as high levels of responsibility. These aspects contribute to emotional exhaustion, impacting on mental health and contributing to poor sleep patterns, anxiety and the use of negative coping strategies such as drugs [85]. However, no interactive effects of emotional exhaustion were observed, only main effects. According to the COR-model, the leaving intentions of the professionals would appear to be a path to recovery in the face of emotional exhaustion. Moreover, according to this model, the inadequacy of resources initiates a spiral of losses. In this sense, the development of emotional exhaustion and demotivation at work can lead to lower levels of subjective vitality.

With respect to sociodemographic variables and time as controls in the proposed models, positive principal effects appear with intention to leave for older professionals, along with an increased impact as a week progresses. Older professionals seem more likely to question their permanence in the profession, experiencing high levels of intention to leave. Then as the week goes by these feelings become greater, perhaps because of the larger stress and discomfort caused by the variables analyzed, as well as the difficulty of daily recovery. Besides, positive principal effects are found in subjective vitality depending on profession. It seems that levels of vitality are lower among physicians in comparison with nursing workers (i.e., nurses and nurse aides). This interesting result pointed out the different type of demands that physicians and nursing workers may accomplish, as the lower levels of vitality could be due to a higher mental overload and responsibility placed on physicians during the working hours, that could impact on vitality levels [69]. All in all, this subjective vitality and differences depending on professions should to be in-depth explored, as it influences in professionals’ well-being.

Despite its strengths in terms of statistical power of the relationships and the continuous recording of variable data for five consecutive days, this study is not without limitations. First, we measured all variables only once a day, before bedtime. We chose to do this so that healthcare professionals on different shifts could participate.

Second, our sample consisted of employees who were mostly women. Some results in the literature suggest that women are more affected by the stress of the work-family conflict. In our study, gender was controlled and there were no differences based on gender. However, the reality is that the majority of health professionals are women and in that sense they are representative results. However, we will have to continue investigating with a larger sample of men.

Third, we followed the participants over the course of 1 work week, so the between-level effects, which are supposed to measure the relationship between the general level (of one person compared to the other) of intention to leave and vitality, are based on one week. If this week were unusual (e.g., more or less stress than other weeks), they might not be characteristic. We did, however, control for time pressure in our analyses.

Finally, the measures we have used are subjective measures. Future studies should include objective measures and information from other observers. Nevertheless, subjective measures provide useful information from the population of interest, in this case health professionals, and are therefore useful as a base from which to develop preventive actions.

In addition to overcoming these limitations, future research could consider including HLM linear growth models to determine the role of changes over time across each time point [86], rather than using a daily mean average. In this way, we could to analyze how time interacts with all the predictor-criterion and moderation relationships we test to see if these relationships fluctuate over the course of 5 days.

It would also be interesting to replicate this study during times of high work overload, such as the COVID-19 pandemic that health professionals are currently facing. At this time, work-family conflict and levels of exhaustion and depersonalization may be extreme (e.g., professionals acknowledge being very exhausted and angry with citizens and some patients), which could be diminishing the subjective vitality of these workers, and generating greater intentions to leave the profession, now and when this crisis is finished. Finally, it would also be interesting to analyze the moderating and mediating role of variables such as rumination, psychological distancing and guilt in the relationships analyzed in this study. For example, in the face of the crisis generated by COVID-19, healthcare workers may experience a greater workload, and this may generate greater work-family conflict, greater exhaustion, and greater attitudes of cynicism towards their patients, which could generate guilt through rumination, leading them to leave the profession.

## 5. Conclusions

The main conclusion of this study is that daily work-family conflict and burnout increase intentions to leave the health profession and decrease the daily vitality levels of workers in this sector. In addition, the depersonalization dimension of burnout amplifies the negative effect of the work-family conflict on these indicators of permanence and well-being. The obstacles derived from the work-family conflict are one of the most relevant problems in the context of occupational health. Improving working conditions to provide a safe and decent job, together with organizational policies that permit an equilibrium between personal and family life, are crucial to increase the satisfaction of health professionals, their recovery from stress and their wish to stay in their profession.

## Figures and Tables

**Table 1:** Means, standard deviations, intra-class correlations, Cronbach’s alphas and correlations among variables.

Variables	M	SD	ICC	α	1	2	3	4	5	6	7
1. General leaving intentions	1.90	0.83		0.91	1	−0.23 **	0.12	0.39 **	−0.09	0.50 **	−0.13
2. General subjective vitality	4.53	1.10		0.87		1	−0.36 **	−0.48 **	−0.28 **	−0.33 **	0.52 **
3. Daily work-family conflict	1.01	0.77	0.45	0.79 (0.73–0.84)			1	0.37 **	0.15 *	0.22 **	−0.42 **
4. Daily job emotional exhaustion	2.35	0.83	0.33	0.89 (0.88–0.90)				1	0.16 **	0.49 **	−0.36 **
5. Daily job depersonalization	1.45	0.53	0.33	0.85 (0.79–0.89)					1	0.19 **	−0.11
6. Daily leaving intentions	1.49	0.70	0.79	0.89 (0.86–0.92)						1	−0.20 **
7. Daily subjective vitality	2.98	1.09	0.39	0.83 (0.79–0.87)							1

*Note:* ICC = intraclass correlation; * *p* < 0.05; ** *p* < 0.01.

**Table 2:** Multilevel estimates for models predicting daily leaving intentions (*N* = 56 × 5 days = 280 statistical observations).

Variables	Null Model			Model 1			Model 2			Model 3			Model 4		
	Estimate	SE	*t*	Estimate	SE	*t*	Estimate	SE	*t*	Estimate	SE	*t*	Estimate	SE	*t*
Intercept	1.490	0.085	17.529 ***	1.500	0.069	21.73 ***	1.500	0.069	21.73 ***	1.500	0.069	21.73 ***	1.499	0.068	22.044 ***
Sex				0.013	0.011	1.181	0.013	0.011	1.181	0.013	0.011	1.181	0.013	0.011	1.181
Age				0.017	0.007	2.428 *	0.017	0.007	2.428 *	0.017	0.007	2.428 **	0.017	0.007	2.428 *
Time				0.031	0.014	2.214 **	0.028	0.013	2.154 *	0.026	0.013	2 *	0.030	0.013	2.307 *
Shift				0.071	0.053	1.339	0.071	0.053	1.339	0.072	0.053	1.358	0.069	0.052	1.327
Profession				−0.039	0.143	−0.272	−0.040	0.143	−0.279	−0.038	0.143	−0.265	−0.031	0.142	−0.218
General leaving intention				0.529	0.089	5.943 ***	0.529	0.090	5.877 ***	0.529	0.089	5.943 ***	0.530	0.088	6.022 ***
Daily WF conflict							0.196	0.039	5.025 ***	0.157	0.041	3.829 ***	0.169	0.042	4.024 **
Daily job EE										0.144	0.057	2.526 **	0.153	0.056	2.732 **
Daily job DEP										0.086	0.059	1.458	0.124	0.061	2.033 *
Daily Job EE X WF conflict													0.028	0.104	0.269
Daily job D X WF conflict													0.251	0.103	2.437 **
−2 X Log(lh)	309.577			252.129			228.933			219.514			213.166		
Difference of −2 X Log				57.448 ***			23.196 ***			9.419 **			6.348 *		
df				6			1			2			2		
Level 1 intercept variance (SE)	0.099 (0.010)			0.098 (0.010)			0.087 (0.009)			0.083 (0.008)			0.081 (0.008)		
Level 2 intercept variance (SE)	0.384 (0.076)			0.217 (0.047)			0.219 (0.047)			0.219 (0.047)			0.214 (0.046)		

*Note:* WF = Work-family; EE = Emotional Exhaustion; DEP = Depersonalization; Sex is coded as 1 = men, 2 = woman; Profession is coded as 1 = physician, 2 = nursing worker; * *p* < 0.05; ** *p* < 0.01; *** *p* < 0.001.

**Table 3:** Multilevel estimates for models predicting daily subjective vitality (*N* = 56 × 5 days = 280 statistical observations).

Variables	Null Model			Model 1			Model 2			Model 3			Model 4		
	Estimate	SE	*t*	Estimate	SE	*t*	Estimate	SE	*t*	Estimate	SE	*t*	Estimate	SE	*t*
Intercept	2.986	0.122	24.475 ***	3.031	0.094	32.24 ***	3.031	0.093	32.59 ***	3.031	0.094	32.24 ***	3.025	0.094	32.18 ***
Sex				0.003	0.014	0.214	0.003	0.014	0.214	0.003	0.014	0.214	0.004	0.014	0.285
Age				−0.014	0.010	−1.4	−0.014	0.010	−1.4	−0.014	0.010	−1.4	−0.013	0.010	−1.3
Time				0.004	0.030	0.133	0.012	0.028	0.428	0.021	0.027	0.778	0.014	0.027	0.518
Shift				−0.019	0.074	−0.256	−0.019	0.074	−0.256	−0.019	0.074	−0.256	−0.016	0.073	−0.219
Profession				−0.530	0.196	−2.704 ***	−0.530	0.195	−2.704 ***	−0.532	0.196	−2.714 ***	−0.532	0.195	−2.728 ***
General subjective vitality				0.549	0.087	6.310 ***	0.549	0.087	6.310 ***	0.548	0.087	6.298 ***	0.538	0.087	6.183 ***
Daily WF conflict							−0.517	0.082	−6.305 ***	−0.410	0.085	−4.823 ***	−0.446	0.087	−5.126 ***
Daily job EE										−0.410	0.117	−3.504 ***	−0.431	0.116	−3.715 ***
Daily job DEP										0.124	0.122	1.016	0.058	0.125	0.464
Daily job EE X WF conflict													0.075	0.210	0.357
Daily job D X WF conflict													−0.526	0.211	−2.492 **
−2 X Log(lh)	663.542			582.365			545.852			533.605			527.465		
Difference of −2 X Log				81.177 ***			36.513 ***			12.247 ***			6.140 *		
df				6			1			2			2		
Level 1 intercept variance (SE)	0.435 (0.042)			0.453 (0.046)			0.377 (0.038)			0.353 (0.036)			0.344 (0.035)		
Level 2 intercept variance (SE)	0.747 (0.159)			0.349 (0.088)			0.361 (0.088)			0.370 (0.088)			0.364 (0.087)		

*Note*: WF = Work-family; EE = Emotional Exhaustion; DEP = Depersonalization; Sex is coded as 1 = men, 2 = woman; Profession is coded as 1 = physician, 2 = nursing worker; * *p* < 0.05; ** *p* < 0.01; *** *p* < 0.001.

## Data Availability

The data presented in this study are available on request from the corresponding author.
